# Comparison of Neurite Orientation Dispersion and Density Imaging and Two-Compartment Spherical Mean Technique Parameter Maps in Multiple Sclerosis

**DOI:** 10.3389/fneur.2021.662855

**Published:** 2021-06-14

**Authors:** Daniel Johnson, Antonio Ricciardi, Wallace Brownlee, Baris Kanber, Ferran Prados, Sara Collorone, Enrico Kaden, Ahmed Toosy, Daniel C. Alexander, Claudia A. M. Gandini Wheeler-Kingshott, Olga Ciccarelli, Francesco Grussu

**Affiliations:** ^1^Department of Neuroinflammation, Faculty of Brain Sciences, Queen Square Multiple Sclerosis (MS) Centre, University College London (UCL) Queen Square Institute of Neurology, University College London, London, United Kingdom; ^2^Addenbrooke's Hospital, Cambridge, United Kingdom; ^3^Department of Medical Physics and Biomedical Engineering, Centre for Medical Image Computing, University College London, London, United Kingdom; ^4^e-Health Centre, Universitat Oberta de Catalunya, Barcelona, Spain; ^5^Department of Computer Science, Centre for Medical Image Computing, University College London, London, United Kingdom; ^6^Great Ormond Street Institute of Child Health, University College London, London, United Kingdom; ^7^National Institute for Health Research (NIHR) University College London Hospitals Biomedical Research Centre, London, United Kingdom; ^8^Department of Brain and Behavioral Sciences, University of Pavia, Pavia, Italy; ^9^Brain Magnetic Resonance Imaging (MRI) 3T Research Centre, Istituto di Ricovero e Cura a Carattere Scientifico (IRCCS) Mondino Foundation, Pavia, Italy; ^10^Radiomics Group, Vall d'Hebron Institute of Oncology, Vall d'Hebron Barcelona Hospital Campus, Barcelona, Spain

**Keywords:** multiple sclerosis, spherical mean technique, neurite orientation dispersion and density imaging, MNI space, diffusion MRI, microstructure

## Abstract

**Background:** Neurite orientation dispersion and density imaging (NODDI) and the spherical mean technique (SMT) are diffusion MRI methods providing metrics with sensitivity to similar characteristics of white matter microstructure. There has been limited comparison of changes in NODDI and SMT parameters due to multiple sclerosis (MS) pathology in clinical settings.

**Purpose:** To compare group-wise differences between healthy controls and MS patients in NODDI and SMT metrics, investigating associations with disability and correlations with diffusion tensor imaging (DTI) metrics.

**Methods:** Sixty three relapsing-remitting MS patients were compared to 28 healthy controls. NODDI and SMT metrics corresponding to intracellular volume fraction (v_in_), orientation dispersion (ODI and ODE), diffusivity (D) (SMT only) and isotropic volume fraction (v_iso_) (NODDI only) were calculated from diffusion MRI data, alongside DTI metrics (fractional anisotropy, FA; axial/mean/radial diffusivity, AD/MD/RD). Correlations between all pairs of MRI metrics were calculated in normal-appearing white matter (NAWM). Associations with expanded disability status scale (EDSS), controlling for age and gender, were evaluated. Patient-control differences were assessed voxel-by-voxel in MNI space controlling for age and gender at the 5% significance level, correcting for multiple comparisons. Spatial overlap of areas showing significant differences were compared using Dice coefficients.

**Results:** NODDI and SMT show significant associations with EDSS (standardised beta coefficient −0.34 in NAWM and −0.37 in lesions for NODDI v_in_; 0.38 and −0.31 for SMT ODE and v_in_ in lesions; *p* < 0.05). Significant correlations in NAWM are observed between DTI and NODDI/SMT metrics. NODDI v_in_ and SMT v_in_ strongly correlated (*r* = 0.72, *p* < 0.05), likewise NODDI ODI and SMT ODE (*r* = −0.80, *p* < 0.05). All DTI, NODDI and SMT metrics detect widespread differences between patients and controls in NAWM (12.57% and 11.90% of MNI brain mask for SMT and NODDI v_in_, Dice overlap of 0.42).

**Data Conclusion:** SMT and NODDI detect significant differences in white matter microstructure between MS patients and controls, concurring on the direction of these changes, providing consistent descriptors of tissue microstructure that correlate with disability and show alterations beyond focal damage. Our study suggests that NODDI and SMT may play a role in monitoring MS in clinical trials and practice.

## Introduction

Over the last 20 years, diffusion tensor imaging (DTI) (1) has established itself as a modality of choice for imaging the cerebral white matter (WM) with a view to gaining an understanding of its microstructure in health and disease. DTI relies on the hypothesis of Gaussian diffusion to evaluate an effective diffusion tensor for each voxel in an image, from which a number of rotationally-invariant scalar descriptors are obtained, such as fractional anisotropy (FA), a measure of diffusion direction dependence, as well as the mean diffusivity (MD) (2), quantifying the overall amount of diffusion in a voxel.

DTI is a rapid and robust technique that has proven useful to assess microstructural damage in a number of conditions (3). Nonetheless, it is limited by the assumptions that underlie its model (4). Firstly, DTI does not account for partial volume effects, an issue at boundaries between different tissue types where voxels contain a mixture of grey matter, white matter, and cerebrospinal fluid. Secondly, DTI metrics lack specificity to different neuropathological substrates. For example, in white matter, diffusivity is generally significantly larger parallel to axons rather than perpendicular to them producing a large FA. However, this anisotropy can be reduced either by a reduction in the density of axons, an increase in their orientation dispersion, or a combination of both. Lastly, the core assumption of DTI, that of Gaussian diffusion, does not necessarily hold in many areas of white matter at high b-values, due to the restriction to water movement created by cell membranes and in regions of crossing fibres.

Several multi-compartment diffusion MRI approaches have been developed in the past 15 years to increase the specificity of techniques such as DTI to key pathophysiological processes (5–11). These methods make explicit assumptions on water compartmentalisation in neural tissue to capture salient characteristics of high-order b-value dependence in diffusion-weighted signals, thus accounting for departures from Gaussian diffusion.

Two recent and widely popular such techniques include neurite orientation dispersion and density imaging (NODDI) (12) and the spherical mean technique (SMT) (13). Both can be fitted to the same, clinically feasible, multi-shell scanning protocols using clinical scanners.

NODDI fits diffusion data to a three-compartment tissue model: an intra-neurite compartment, in which diffusion is constrained except along the direction of neurites; an extra-neurite compartment, in which diffusion is Gaussian with hindered diffusion perpendicular to the direction of neurites and a CSF compartment, in which diffusion is Gaussian and isotropic. Within each voxel, the variability of neurite orientations is modelled by a Watson distribution.

SMT is another recent technique for microscopic diffusion anisotropy imaging (13) that maps microstructural tissue features not confounded by fibre crossing or orientation dispersion. In this study, we used an SMT method comprising two tissue compartments (14), i.e., an intra- and an extra-neurite compartment. This approach provides estimates of the neural diffusivity and the intra-neurite volume fraction in the presence of orientation heterogeneity. Subsequently, spherical deconvolution can be used to recover the fibre orientation distribution, from which the orientation dispersion entropy is calculated voxel-by-voxel.

Both techniques were applied to people with Multiple Sclerosis (MS). MS is a disease of the central nervous system with a complex and incompletely understood pathophysiology. It features both an inflammatory demyelinating component, which leads to discrete focal white matter lesions and a neurodegenerative component, resulting in generalised diffuse white and grey matter atrophy (15). Abnormal DTI measures are seen in both lesions and so-called normal appearing white matter (NAWM) (featuring no obvious pathology on conventional MRI scanning) (16–18). There is evidence that validates the use of NODDI in MS (19–21), which suggest that while FA might be the most sensitive metric to detect abnormalities, NODDI metrics are able to detect areas of abnormality where FA is normal (22, 23). Investigations into NODDI in MS have generally concluded that intra-neurite volume fraction decreases in both lesions and NAWM (20, 21, 24) whilst findings on orientation dispersion vary (23, 24). Considering SMT, differences between lesions and NAWM in MS patients but not between NAWM and healthy WM in controls in similar anatomical locations were reported in the brain (25), and abnormalities were also detected in the NAWM of the MS cervical spinal cord (26).

Notably, SMT and NODDI provide metrics with similar biological interpretations that can be obtained from similar diffusion-weighted MRI data. They are promising biomarkers in MS and have the potential of increasing the capacity of MRI to detect MS pathology. Here, for the first time to the authors' knowledge, we directly compare NODDI and SMT metrics in a real clinical setting by analysing the same data, acquired with a multi-shell diffusion weighted MRI protocol, and investigating the ability of NODDI and SMT to detect abnormalities in patients with relapsing-remitting (RR) MS compared with healthy controls, as well as their association to physical disability. Our results are informative for the community and elucidate whether (i) NODDI and SMT detect changes in similar locations and that these approaches point toward the same biological alterations; (ii) are the results derived by the two techniques sufficiently similar as to allow direct cross-comparison; or (iii) are there differences precluding this or potentially highlighting an advantage to one or other method in detecting group differences between MS patients and controls.

## Methods

### Participants

This is a retrospective analysis using data previously acquired, and reported in full elsewhere (27). Sixty-three patients with RRMS (48 female, 76%) and 28 heathy controls (19 female, 68%) were recruited. Mean ages were 47 (SD ± 7.6) years for patients and 35.1 (±10.2) for healthy controls. For RRMS patients, mean disease duration at the time of assessment was 14.6 years (SD ± 2.4) and Expanded Disability Status Scores ranged from 0 to 6.5, with a median score of 2. All subjects provided written informed consent and the study was approved by the institutional Research Ethics Committee.

### MR Image Acquisition

All subjects were scanned with a 3T Philips Achieva MRI system. The scanning protocol included multi-shell diffusion-weighted echo-planar imaging scans (resolution: 2.5 × 2.5 × 2.5mm^3^; TE = 82 ms; TR = 14 s; b = 0 and {8,15,30} directions at b = {300, 711, 2,000} s/mm^2^, scan time 16'34”), anatomical PD-T2 images for MS lesion outlining (multi-slice turbo spin echo; resolution: 1 × 1 × 3 mm^3^, TE = 19/85 ms, TR = 3,500 ms, scan time 4'01”), and volumetric T1-weighted imaging (3D turbo field echo, resolution 1 × 1 × 1 mm^3^, flip angle 8°, TE = 3.1 ms, TR = 6.9 ms, TI = 824 ms, scan time 6'30”).

### Diffusion Metrics Evaluation

The multi-shell diffusion data were corrected for motion and eddy current distortion using the FSL *eddy* (28). Non-brain tissue was eliminated using co-registered T1 images using the FSL Brain Extraction Tool (https://fsl.fmrib.ox.ac.uk/fsl/fslwiki/BET). The DTI model was fitted to the diffusion data with the exclusion of the highest b-value shell to limit the contribution of non-Gaussian diffusion, using FSL *dtifit*. This provided the following voxel-wise metrics: Fractional Anisotropy (FA); axial/radial/mean diffusivities (AD/RD/MD).

Images were analysed using NODDI (12) to obtain maps of orientation dispersion index (ODI), isotropic volume fraction (v_iso_) and intra-neurite volume fraction (v_in_-NODDI). ODI is defined as (2/π)arctan(1/κ) where κ is the width parameter of the Watson distribution describing the neurite orientation distribution. ODI therefore increases with increasing orientation dispersion. The NODDI model was fitted with the freely available Matlab Toolbox (29) (http://mig.cs.ucl.ac.uk/index.php?n=Tutorial.NODDImatlab).

Two-compartment SMT (14) analysis was performed on the same data used for NODDI with the freely available SMT fitting routines (30) (https://github.com/ekaden/smt) and provided voxel-wise intra-neurite volume fraction (v_in_-SMT) and neural diffusivity (D). Additionally, orientation dispersion entropy (ODE) was obtained using in-house Matlab code. ODE is defined as the Kullback-Leibler divergence of the axon orientation distribution with respect to the uniform distribution (14), implying that ODE is reduced as neurite dispersion increases. As a consequence, ODI and ODE show reversed contrast: increasing local neurite orientation variability is mapped to increasing NODDI ODI and decreasing SMT ODE values. The SMT and NODDI parameters and the main model constraints are described and summarised in Table 1. The fitting of both NODDI and SMT was performed using the same procedure (i.e., same fitting code with same constraints) across the whole brain, including lesional voxels. Among NODDI and SMT parameters, the obvious comparisons are between ODE and ODI and the SMT and NODDI intra-neurite volume fraction metrics, as such metrics are designed to capture similar characteristics of tissue microstructure. In the case of ODI and ODE the comparison is less direct with the metrics expected to be approximately inversely proportional to each other. However, as both are measures of the degree of alignment of axons within a voxel, from the perspective of identifying voxels showing a significant difference between MS patients and controls, they are adequately comparable. Finally, we point out that NODDI and SMT metrics of compartment-wise fraction (i.e., v_iso_ and v_in_ for NODDI; v_in_ for SMT) are inherently T2-weighted. Therefore, they technically represent signal fractions, rather than volume fractions. Nonetheless, the latter definition has become more popular in the literature and will be adopted here.

**Table 1:** Summary of the diffusion MRI metrics provided by the NODDI and SMT techniques, as well as overview of the constraints adopted in each of the two models.

**MRI technique and model constraints**	**Metric**	**Description**
NODDI - 3 compartments (free water; intra-/extra-neurite) - Free water diffusivity: 3 μm^2^/ms - Intra-/extra-neurite parallel diffusivity: 1.7 μm^2^/ms - Zero intra-neurite perpendicular diffusivity - Tortuosity model for extra-neurite perpendicular diffusivity - Watson distribution for neurite orientations	Intra-neurite volume fraction (v_in_) Orientation dispersion index (ODI) Isotropic volume fraction (v_iso_)	Fraction of neural tissue signal (i.e., excluding CSF) originating inside axons/dendrites Variability of axon/dendrite orientations (higher ODI implies higher orientation variability) Fraction of total signal originating in free water (e.g., CSF)
Two-compartment SMT - 2 compartments (intra-/extra-neurite) - Intra-/extra-neurite parallel diffusivity: fixed to same value, but estimated - Zero intra-neurite perpendicular diffusivity - Tortuosity model for extra-neurite perpendicular diffusivity - No assumptions on the distribution of neurite orientations	Intra-neurite volume fraction (v_in_) Orientation dispersion entropy (ODE) Neural diffusivity (D)	Fraction of total signal (i.e., including CSF) originating inside axons/dendrites. Note that SMT v_in_ is formally equivalent to NODDI (1 – v_iso_) v_in_ Coherence of axon/dendrite orientations (higher ODE implies lower orientation variability, so ODE ~ 1/ODI) Average neural tissue diffusion coefficient parallel to the local direction of fibres

### Tissue Characterisation

Lesion masks were traced manually by a single rater (WJB) viewing PD/T2-weighted images using a semi-automated edge-finding tool (JIM6.0, Xinapse Systems, UK). Afterwards, PD/T2-weighted images and lesion masks were registered to diffusion space using affine co-registration (NiftyReg *reg_aladin* tool, http://cmictig.cs.ucl.ac.uk/wiki/index.php/NiftyReg). Finally, we lesion-filled the 3D T1-weighted anatomical scan of each patient, co-registered it to diffusion space using affine co-registration with NiftyReg *reg_aladin* and segmented the brain into different tissues with the GIF technique (31). Afterwards, we extracted mean values of NODDI and SMT metrics within each tissue type (white matter lesions, normal appearing white matter, cortical grey matter, deep grey matter) for each patient, and obtained tissue-specific distributions of all NODDI and SMT metrics.

### Analysis in Standard Space

We warped all MRI metrics (DTI, NODDI, SMT), lesion masks and tissue segmentations to the standard MNI152 space (2 mm isotropic resolution; lesions resampled with nearest-neighbour interpolation. For this purpose, we co-registered non-linearly the lesion-filled T1-weighted anatomical scans to the MNI template using NiftyReg *reg_f3d*. Afterwards we combined such a registration transformation to that, warping diffusion data to each subject's T1-weighted scan, and resampled all parametric maps, lesion and tissue masks with NiftyReg *reg_resample*. Firstly, we investigated associations between all possible pairs of MRI metrics by calculating Pearson's correlation coefficients on the subject-wise WM mean values (NAWM for patients, i.e., excluding lesions).

Secondly, we investigated the association between all MRI metrics and disability, as measured by the EDSS score. We fitted the model *edss* = β_0_+ β_1_*m*+ β_2_*age*+ β_3_*gender* for each MRI metric *m* among FA, AD, RD, MD (DTI); v_iso_, v_in_, ODI (NODDI); D, v_in_, ODE (SMT). The models, which consider age and gender as confounding factors, where fitted with the python *statsmodel* module twice for each metric: in one case using metric mean value in patients' NAWM, and in the second case using lesions' mean values.

Afterwards, we tested whether MRI metrics exhibited differences between RRMS patient and controls. For this purpose, we fitted voxel-by-voxel the model *m* = β_0_+ β_1_*group*+ β_2_*age*+ β_3_*gender* with the freely available python *statsmodel* module. Above, *m* is the generic MRI metric (FA, AD, RD, MD for DTI; v_iso_, v_in_, ODI for NODDI; D, v_in_, ODE for SMT). The models account for age and gender as confounding factors, and were fitted excluding measurements from lesional voxels, i.e., including only normal-appearing tissue. In practise, this was achieved by excluding patients whose resampled lesion mask included a specific voxel when fitting our regression model in that voxel. The *p*-value maps corresponding to the coefficients β_0_, β_1_, β_2_, and β_3_ were corrected for multiple comparisons with FSL *fdr*. An MRI metric was considered significantly different between patients and controls in a voxel if a non-zero β_1_ showed a *p* < 0.05 (correcting for multiple comparisons).

Finally, we calculated the volume of the tissue in MNI space exhibiting statistically significant patient-control differences, and quantified the overlap among such areas for all possible pairs of MRI metrics by computing the Dice overlap coefficient (32).

## Results

### Examples of Lesion Segmentation

**Figure 1:**
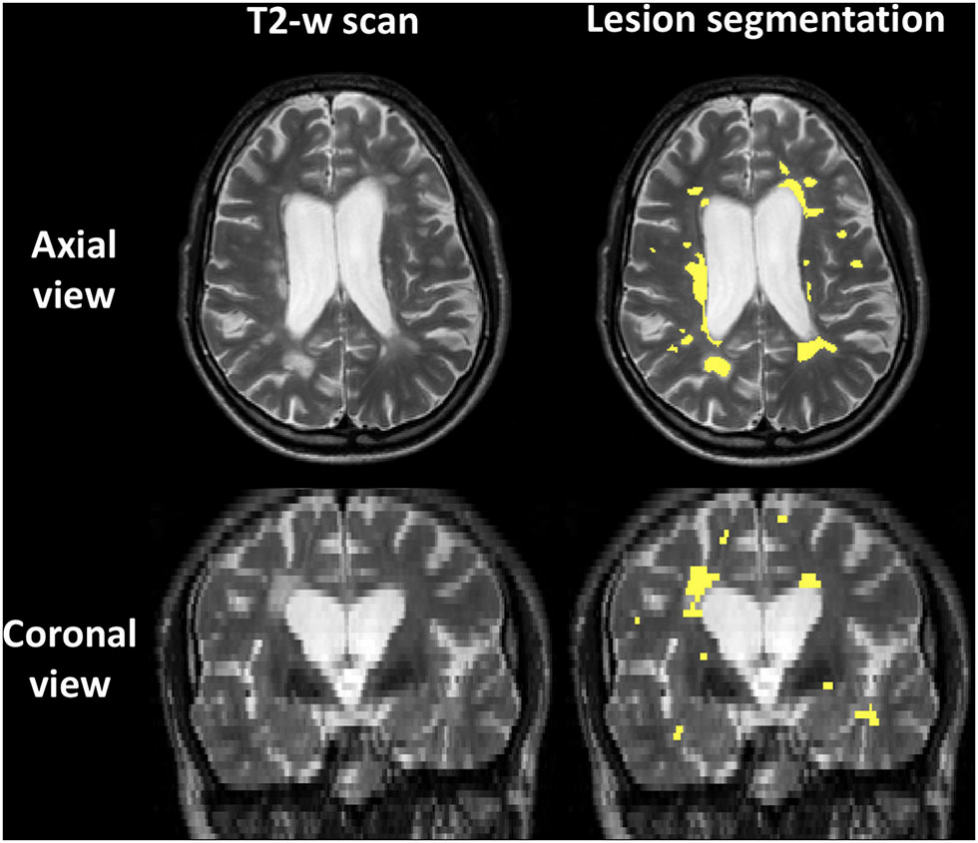
Example of lesion segmentation on the T2-weighted fast spin echo 2D anatomical scan of one patient. **Top**: axial views; **bottom**: coronal views. **Left**: scan; **right**: scan with lesions outlined in yellow.

Figure 1 shows an example of lesion segmentation in one patient. Segmentation was performed on the PD-weighted/T2-weighted axial turbo spin echo scans, which demonstrate WM lesions as hyperintense as compared to the surrounding normal-appearing tissue.

### Examples of Parametric Maps

**Figure 2:**
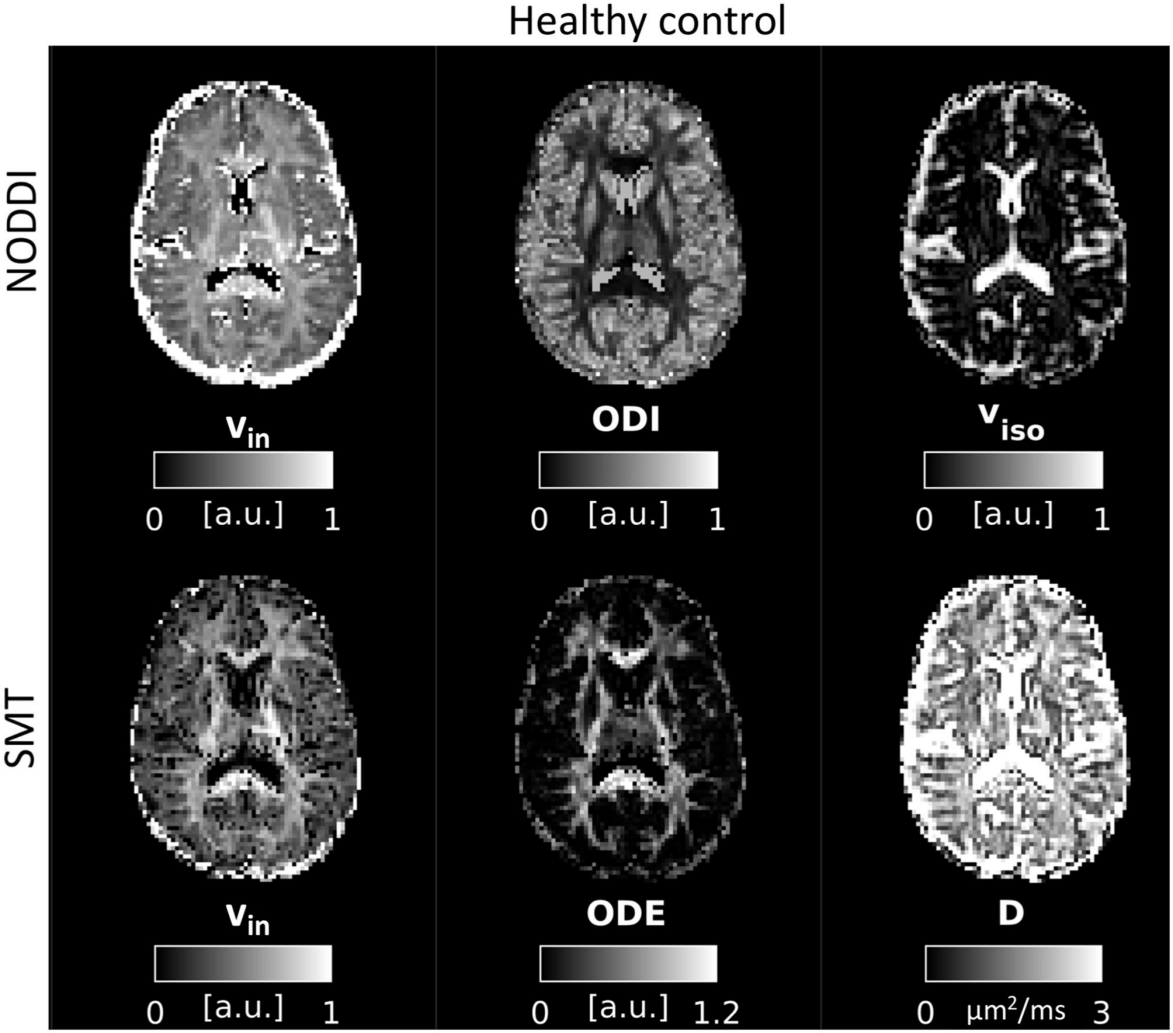
NODDI and SMT metrics as obtained from a healthy control. **Top**: NODDI (**left**: intra-neurite volume fraction v_in_; orientation dispersion index ODI; isotropic volume fraction v_iso_); **bottom**: SMT (intra-neurite volume fraction v_in_; orientation dispersion entropy ODE; neural diffusivity D).

**Figure 3:**
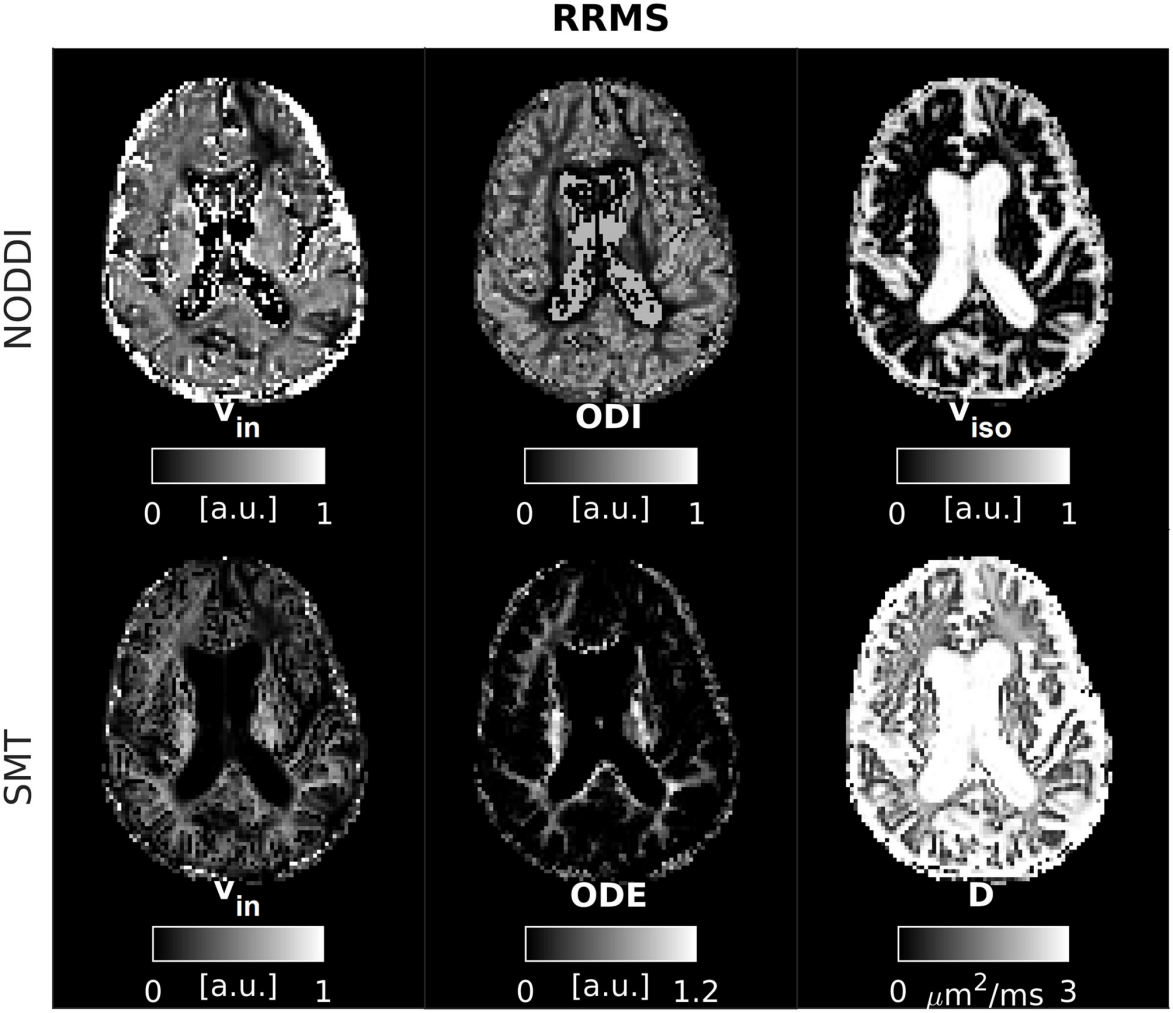
NODDI and SMT metrics as obtained from an MS patient. **Top**: NODDI (**left**: intra-neurite volume fraction v_in_; orientation dispersion index ODI; isotropic volume fraction v_iso_); **bottom**: SMT (intra-neurite volume fraction v_in_; orientation dispersion entropy ODE; neural diffusivity D).

An example of parametric NODDI and SMT maps from a healthy control and an RRMS patient are provided in Figures 2, 3, respectively. Visual inspection highlights known between-tissue contrasts, e.g., higher NODDI and SMT v_in_ in WM than in GM, lower/higher NODDI ODI/SMT ODE in GM than in WM (control); hypointense lesions in both NODDI and SMT v_in_ maps (patient).

### Tissue-Specific Distributions and Correlations

Supplementary Figure 1 shows tissue-specific distributions of all NODDI and SMT metrics. Between-tissue contrasts are apparent in all metrics, and agree well with previously reported numerical values (21, 25). NODDI v_in_ and NODDI (1 – v_iso_)v_in_ show similar between-tissue contrasts as compared to SMT v_in_, while NODDI ODI show between-tissue contrasts that follow opposite directions as compared to SMT ODE. Numerical values of both NODDI v_in_ and NODDI (1 – v_iso_)v_in_ are qualitatively higher than SMT v_in_ in all tissues.

**Figure 4:**
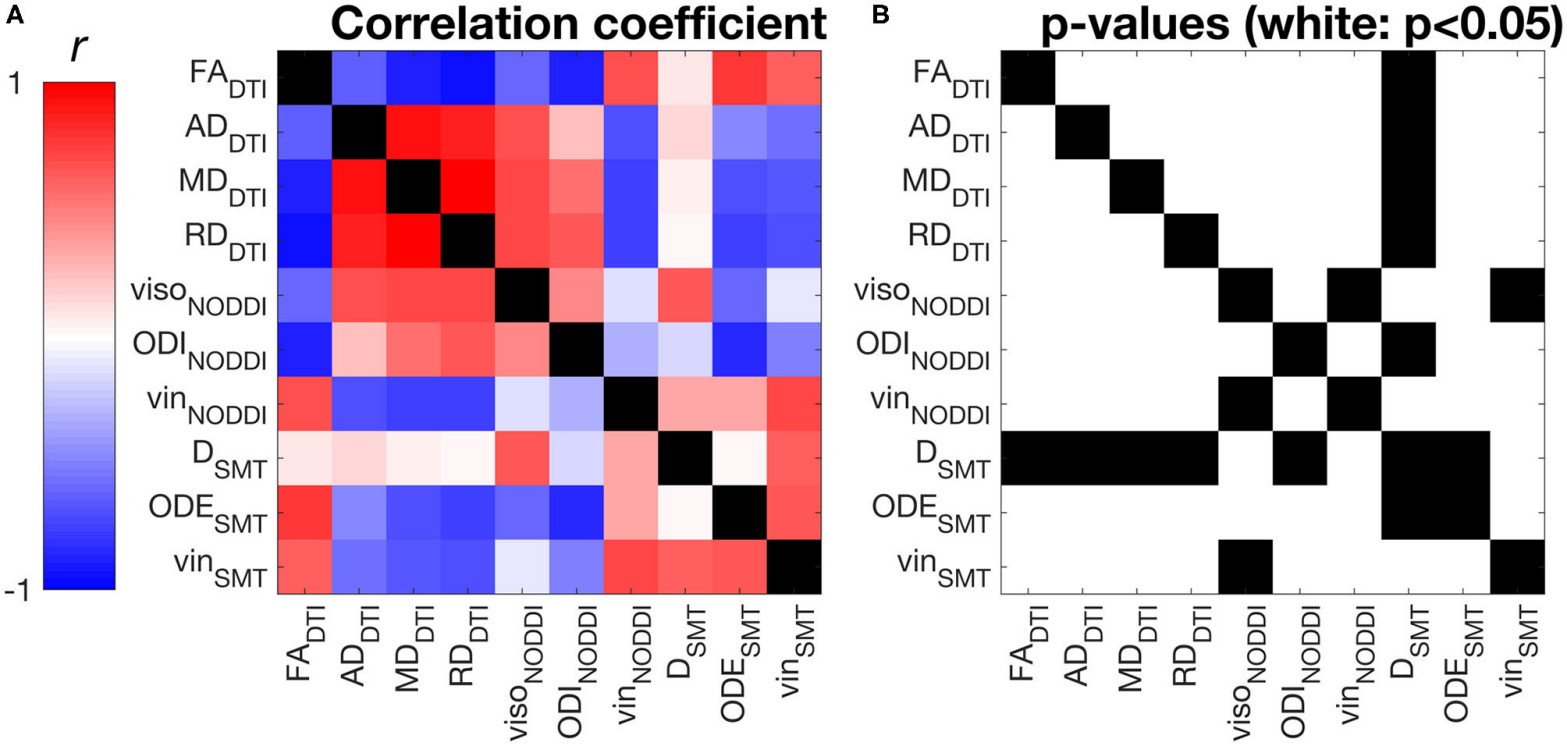
Pearson's linear correlation among all possible pairs of MRI metrics from DTI, NODDI and SMT. **(A)** correlation values illustrated as a 2D symmetric matrix (note its symmetry with respect to the diagonal) and calculated using the normal-appearing white matter (NAWM) mean values of the metrics (white matter for controls). **(B)** statistically significant correlations, highlighted in white (*p* < 0.05).

Figure 4 shows correlations between all possible pairs of MRI metrics, as evaluated on WM values (WM for controls, NAWM for patients, i.e., excluding lesions). NODDI and SMT metrics exhibit strong correlations with DTI indices, as well as with themselves. For example, NODDI v_iso_ is positively associated with all DTI diffusivities and with SMT neural tissue diffusivity D. NODDI v_in_ is positively associated with FA and negatively associated with DTI AD, RD MD, while the opposite holds for ODI. For SMT, D is positively associated to NODDI v_iso_, while both SMT ODE and v_iso_ are positively associated with DTI FA and negatively with DTI diffusivities. The strongest correlation for NODDI v_in_ is with SMT v_in_ (*r* = 0.72, *p* < 0.05), with NODDI v_in_ also being the strongest correlate for SMT v_in_. The strongest correlates of NODDI ODI are DTI FA and SMT ODE (*r* = −0.83 with FA and *r* = −0.80 with ODE, *p* < 0.05). FA and NODDI ODI are the strongest correlates of SMT ODE (*r* = −0.78 with FA and *r* = −0.80 with ODE, *p* < 0.05).

### Association With Disability

Table 2 shows the association between EDSS and DTI, NODDI and SMT metric mean values in NAWM and within lesional WM. The table reports standardised coefficients, which are therefore comparable across metrics (the closer to 1 in absolute value, the stronger the association with EDSS). In NAWM, we observe significant associations for DTI metrics (negative association for FA; positive association for diffusivities) and NODDI (negative association for v_in_). In lesions, similar results are seen for DTI (negative association for FA; positive association for MD and RD) and NODDI (negative association for v_in_), while in this case also observe associations for SMT (positive association for ODE, negative for v_in_). DTI indices show the strongest association with EDSS in both NAWM and lesional WM.

**Table 2:** Association between EDSS and DTI, NODDI and SMT metrics as obtained from linear regression models.

		**DTI metrics**				**NODDI metrics**			**SMT metrics**		
		**FA**	**AD**	**MD**	**RD**	**v_**iso**_**	**ODI**	**v_**in**_**	**D**	**ODE**	**v_**in**_**
NAWM	Coefficient	−0.33 (0.15)	0.35 (0.15)	0.35 (0.14)	0.35 (0.14)	0.25 (0.15)	0.10 (0.15)	−0.34 (0.15)	0.13 (0.16)	−0.28 (0.15)	−0.22 (0.16)
	*p*-value	0.027*	0.020*	0.016*	0.018*	0.105	0.532	0.025*	0.431	0.065	0.170
WM lesions	Coefficient	−0.47 (0.15)	0.25 (0.15)	0.36 (0.15)	0.41 (0.15)	0.16 (0.16)	0.09 (0.15)	−0.37 (0.15)	−0.03 (0.16)	0.38 (0.15)	−0.31 (0.15)
	*p*-value	0.003*	0.100	0.020*	0.010*	0.313	0.546	0.018*	0.828	0.016*	0.043*

The table reports the estimate, standard error (in brackets) and p-values of the coefficient β_1_ in the model edss = β_0_+ β_1_m+ β_2_age+ β_3_gender controlling for age and gender, with m being the generic MRI metric of interest. The models were fitted to the normal-appearing white matter (NAWM) mean values of the MRI metrics, as well as to the white matter (WM) lesion mean values. p < 0.05 is flagged by an asterisk (^*^). The table reports coefficients β_1_as standardised, implying that they are comparable across different MRI metrics.

### Group Comparison

**Figure 5:**
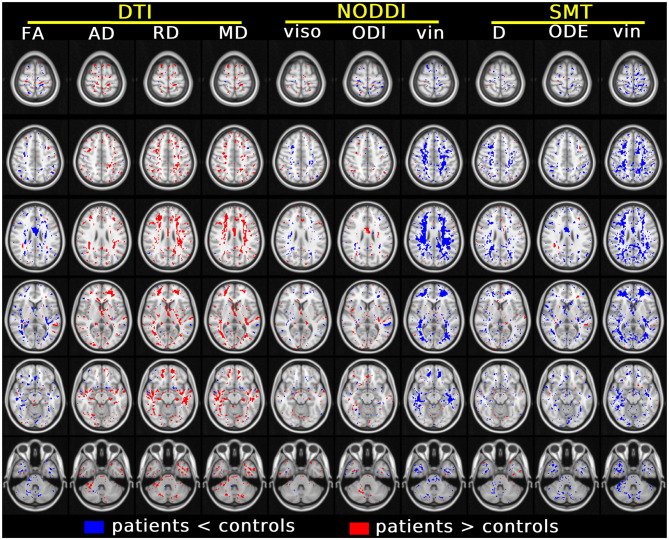
Results of the voxel-wise group comparison performed in MNI space. The figure visualises voxels at six different axial levels where a specific MRI metric from DTI, NODDI and SMT differs significantly between patients and controls (blue/red: metric from patients smaller/larger than controls). The model used for this comparison, in the form of *m* = β_0_+ β_1_*group*+ β_2_*age*+ β_3_*gender* with *m* being the generic MRI metric, adjusts for age and gender. In each MNI voxel, the model was fitted excluding measurements from focal lesions (i.e., including only normal-appearing tissues). A threshold of *p* < 0.05, correcting for multiple comparisons with the FSL *fdr* tool, was chosen for statistical significance. The figure shows voxels where β_1_ is statistically different from 0.

Figure 5 shows the results of the voxel-wise patient-control comparison in MNI space. The figure illustrates voxels where significant differences between patients and controls are detected, colouring them according to the sign of the difference. Patient-control differences are observed mainly at the level of cerebral WM, although some scattered voxels are also observed in GM and in the cerebellum. For DTI, it is seen that FA is in general reduced in patients compared to controls, while AD, RD and MD are generally increased, although in some regions small clusters of decreased diffusivities are also seen. Both increases and decreases of NODDI v_iso_ and ODI and SMT D and ODE are seen, while both NODDI v_in_ and SMT v_in_ show extensive, widespread reductions in patients compared to controls.

Table 3 reports the volume of the tissue in MNI space where significant patient-control differences were detected for all MRI metrics. The three metrics that detect differences in the largest portions of tissue are SMT v_in_ (229.8 ml) and NODDI v_in_ (217.5 ml) and DTI RD (141.5 ml). The three metrics that detect differences in the least volume of tissue are instead SMT ODE (87.4 ml), NODDI ODI (67.1 ml) and, above all, NODDI v_iso_ (62.0 ml).

**Table 3:** Volume of tissue in MNI space exhibiting statistically significant differences between patients and controls for all MRI metrics (DTI, NODDI, and SMT).

	**DTI metrics**				**NODDI metrics**			**SMT metrics**		
	**FA**	**AD**	**MD**	**RD**	**v_**iso**_**	**ODI**	**v_**in**_**	**D**	**ODE**	**v_**in**_**
Volume (ml)	106.8	92.9	142.1	141.5	62.0	67.1	217.5	98.5	87.4	229.8
Fraction of brain mask	5.84%	5.08%	7.77%	7.74%	3.39%	3.67%	11.90%	5.39%	4.78%	12.57%

Finally, Table 4 reports the spatial overlap of the areas where significant differences are detected, considering all possible pairs of MRI metrics. The largest overlap is seen for DTI FA and MD (0.71). For NODDI v_in_, the largest overlap is seen with SMT v_in_ (0.42), while for SMT v_in_ the largest overlap is the NODDI v_in_. For NODDI ODI, the largest overlaps are with DTI FA (0.36) and SMT ODE (0.31). Findings were similar for SMT ODE (overlap of 0.40 with FA and 0.31 with ODI). NODDI v_iso_ and SMT D are each the other's largest overlap (0.42).

**Table 4:** Extent of the spatial overlap, measured by the Dice coefficient (0: no overlap; 1: full overlap), among areas exhibiting statistically significant patient-control differences for all MRI metrics (DTI, NODDI, and SMT).

	**DTI metrics**				**NODDI metrics**			**SMT metrics**		
	**FA**	**AD**	**MD**	**RD**	**v_**iso**_**	**ODI**	**v_**in**_**	**D**	**ODE**	**v_**in**_**
DTI FA	–	0.09	0.24	0.43	0.08	0.36	0.24	0.13	0.40	0.23
DTI AD	–	–	0.46	0.32	0.19	0.12	0.15	0.11	0.09	0.14
DTI MD	–	–	–	0.71	0.18	0.11	0.38	0.15	0.14	0.29
DTI RD	–	–	–	–	0.17	0.18	0.38	0.16	0.25	0.32
NODDI v_iso_	–	–	–	–	–	0.06	0.13	0.42	0.06	0.14
NODDI ODI	–	–	–	–	–	–	0.11	0.08	0.31	0.11
NODDI v_in_	–	–	–	–	–	–	–	0.25	0.11	0.42
SMT D	–	–	–	–	–	–	–	–	0.08	0.34
SMT ODE	–	–	–	–	–	–	–	–	–	0.23
SMT v_in_	–	–	–	–	–	–	–	–	–	–

## Discussion

### Summary

We have investigated the ability of two diffusion MRI-based techniques, NODDI and two-compartment SMT, to detect group differences between relapsing remitting MS and healthy controls as well as the association of their metrics to EDSS, a measure of physical disability. NODDI and SMT provide metrics with similar biological interpretation, obtained in different ways (i.e., different model assumptions and different parameter estimation approaches). To our knowledge this is the first time that the two techniques have been compared directly on the same MRI data acquired on a 3T clinical scanner in healthy controls and patients with MS.

Our main findings are that both NODDI and SMT detected differences in microstructure between MS patients and healthy controls. The two techniques provide metrics with similar biophysical interpretation that correlate strongly with each other. They generally concur on the nature of alterations seen to MS pointing toward similar pathological changes. Interestingly, SMT detected significant difference in patients vs. controls comparisons in a slightly higher proportion of voxels as compared to NODDI. Finally, the two techniques provide metrics that show an association to physical disability when evaluated across NAWM tissue (NODDI v_in_) and lesional WM (NODDI v_in_ and SMT ODE and v_in_).

### DTI Results

In this study we have considered DTI indices, namely FA, AD, RD and MD, as these provide a well-established reference to which the performance of NODDI and SMT can be compared. Our results concur with DTI's known sensitivity to MS pathology. DTI metrics reveal widespread differences in normal-appearing tissues between patients and controls, and show the strongest association to EDSS. All DTI diffusivities (e.g., AD, RD and MD) generally show an increase in patients compared to controls. This is in line with recent literature. For instance, in (19) increased DTI axial diffusivity *ex vivo* was seen, which was driven by a strong demyelination as seen in histology. In (33) increases in AD was seen in MS lesions as compared to corresponding healthy tissue in controls, while in (34) both increases and decreases of AD in the same cohort of MS patients were observed, compared to healthy controls.

### NODDI Results

Our data is generally consistent with previous findings. For intra-neurite volume fraction v_in_, our results point toward reduction in this metric in both lesions and NAWM in RRMS patients compared with healthy controls. Such reductions are consistent with previous findings (20, 21, 23, 24), and may be indicative of diffuse axonal loss as well as secondary demyelination (19).

We also report changes in ODI in both NAWM and within lesions. Previous studies reported both increases and decreases of ODI in lesions (20, 23, 24). A possible explanation for such mixed results rests in the observation that studies reporting a decrease have either looked at lesions individually, comparing with healthy controls at the same anatomical location or may have been limited by smaller sample sizes. In addition, T2-hyperintense lesions possess pathological heterogeneity, ranging from active inflammation displaying gadolinium-enhancement, to T1-hypointense “black holes” and therefore have different underlying tissue microstructures. Here we detect changes in ODI that go in both directions (i.e., both increased and decreased ODI values as compared to controls). This suggests that in MS pathology it is possible that neurites can become either more coherently aligned or more dispersed as compared to healthy tissue, possibly reflecting different pathological mechanisms.

### SMT Results and Comparison

Previous SMT work has focussed on intra-neurite volume fraction only and has reported reductions in white matter lesions in both the cervical spine (26) and cerebral white matter (25). A reduction in intra-neurite volume fraction in NAWM compared with healthy controls is seen in the cervical spine (26) but not in cerebral NAWM (25). We generally concur with these findings, although the discrepancy over intra-neurite volume fraction in NAWM may arise from differences between a region-of-interest (ROI) based approach, confined to the internal capsule, compared with our whole-brain, voxel-wise evaluation. Quantitative values of NODDI intra-neurite volume fraction v_in_ appear qualitatively higher than SMT v_in_ (Supplementary Figure 1). A similar trend is observed even after correcting NODDI v_in_ for isotropic water partial volume (which is not modelled in SMT), i.e., when comparing regional values of NODDI (1 – v_iso_)v_in_ with SMT v_in_.

We observe increases and decreases in ODE that concur with the direction of the change of NODDI ODI (albeit with opposite sign, given the different metric definition). This finding gives confidence about the potential existence of multiple patterns of alteration of neurite fibre dispersion in MS, being observed in two independent techniques (NODDI and SMT).

### Group Comparison

We compared values of all MRI metrics from DTI, NODDI and SMT between patients and controls in MNI space, excluding lesional voxels (i.e., considering only normal-appearing tissue). Our results demonstrate that all of DTI, NODDI and SMT detect widespread differences in normal-appearing tissue between the cohorts, implying that these techniques may play a role in the assessment of MS damage beyond focal lesions.

NODDI and SMT detect group-wise differences between MS patients and controls that go in the same direction and point toward the same underlying pathophysiological features. Our results also show that SMT detected alterations in a slightly larger portion of the brain compared to equivalent NODDI-derived metrics. We speculate that this difference may arise from the lower number of model assumptions in SMT compared with NODDI (e.g., no fixed value for average neural diffusivity; no fixed form for the neurite orientation distribution), or could be a secondary effect due to the lack of a third isotropic diffusion compartment in the signal model, which can capture several sources of local free water contamination. Moreover, our analysis shows that both increases and decreases in dispersion metrics (i.e., NODDI ODI and SMT ODE), NODDI isotropic volume fraction v_iso_ and SMT neural diffusivity D can be observed in MS patients compared with healthy controls.

Areas of increased v_iso_ and D in MS patients may be a secondary effect of atrophy, particularly where adjacent to CSF spaces, resulting from partial volume effects occurring more frequently in patients. Decreased SMT D is in general not mirrored by DTI diffusivities (AD, RD and MD). DTI diffusivities are known to be influenced by the underlying dispersion of neural fibres, and effect that by construction should be less strong on SMT D (as fibre dispersion effects are captured by SMT ODE). This implies that that D may capture alterations in diffusivity that are unconfounded by changes in orientation dispersion, unlike DTI diffusivities. Nonetheless, both SMT D and NODDI v_iso_ should be interpreted with considerable care. Estimating the intrinsic diffusivity of neural tissue is known to be a challenging task, and v_iso_ is known to be poorly reproducible and confounded by relaxation effects, being heavily T2-weighted (35), which may explain the high v_iso_ (up to 0.2) seen here in control WM. Finally, it should be remembered that NODDI and SMT are likely to exhibit different susceptibility to noise given their very different fitting strategies. This may affect the estimation of metrics such as v_iso_ and D. In future, we plan to employ computer simulation to characterise extensively the susceptibility to noise of the two techniques. Moreover, we remark that extensive histological validation based on multiple stainings in health and disease will be required for metrics such as NODDI v_iso_ and SMT D to elucidate the relationship with the underlying pathophysiology, given the lack of a direct, histological counterpart.

Importantly, it should be noted that in our case-control comparison, classes were slightly imbalanced (i.e., our cohort features approximately twice as many patients as controls). Tests show that the differences detected in this study are greatly preserved after matching the two group sizes by downsampling the patient group (Supplementary Figure 2). This confirms that the conclusions of this paper still hold despite the imbalance of our data set. In future, we will aim for a control group that more closely matches the characteristics of the patients' group.

### Association With Disability

Having established the presence of group differences between MS patients and controls, we investigated the relationship between NODDI and SMT metrics and physical disability, as measured by the EDSS score. We used a linear regression model, adjusting for age and gender, to study the association between mean values of NODDI and SMT metrics obtained from NAWM and lesional tissue in standard MNI space. We detect a significant association between NODDI v_in_ in NAWM and EDSS. We also detect a significant association between SMT v_in_ and ODE mean values in WM lesions and EDSS. These results suggest that novel multi-shell multi-compartment models (e.g., NODDI and SMT) may be able to detect early, widespread alteration in axonal density and/or myelination (note that v_in_ is sensitive to both) that play a crucial role in disability accumulation. Moreover, they may also be able to give new insight into the microstructural characteristics of existing focal damage, potentially providing useful information in practise settings to aid in stratifying patients, potentially and guiding treatment selection and prognosis. Our NODDI and SMT findings agree with previous literature, with a number of studies having reported the potential of microstructural diffusion MRI techniques to detect MS pathology and association to disability beyond focal damage (36, 37). They are also confirmed by DTI metrics, which exhibit strong associations with physical disability in our RRMS cohort (somewhat stronger than NODDI and SMT). All in all, our results point toward the utility of diffusion imaging in the MS clinic alongside routine anatomical imaging, which is crucial for accurate MS lesion detection. Lesion distribution heterogeneity may carry important clinical information *per se*, and could therefore provide descriptors of MS pathology that are complementary to the diffusion metrics focus of this study.

### Diffusion MRI Metrics Correlation

In this study we have investigated the correlation among all possible pairs of diffusion MRI metrics considered in this study (DTI, NODDI and SMT metrics). The analysis shows that indices of intra-neurite volume fractions v_in_, derived independently from NODDI and SMT, correlate strongly with each other (Figure 4). Considering orientation dispersion metrics, it should be noted that NODDI ODI and SMT ODE measure different features of the underlying neurite orientation distributions, and therefore are not modelling the same physical quantity (Figure 4). Nonetheless, qualitative inspection of tissue-specific distributions (Supplementary Figure 1) and correlation analyses show that the information provided by ODI and ODE mirror each other. The two metrics appear approximately inversely proportional to each other and exhibit a strong negative linear correlation. Overall, our comparison suggests that NODDI and SMT metrics carry very similar information and are therefore likely to offer highly comparable descriptions of the underling tissue microstructure.

Moreover, our correlation analysis also demonstrates that NODDI and SMT are strongly correlated to DTI indices (e.g., strong, positive correlation between DTI FA and both NODDI v_in_ and SMT v_in_; negative/positive correlation between DTI FA and NODDI ODI/SMT ODE). This finding can be understood by noting that metrics provided by multi-compartmental techniques such as NODDI and SMT are not only sensitive to the non-Gaussian characteristics of the diffusion process (emphasised at high b-value), but also to the Gaussian characteristics, i.e., on the diffusion tensor and on its metrics (e.g., FA, AD, RD and MD) (38). Nonetheless, we remark that methods such as NODDI and SMT aim to disentangle the different pathophysiological processes that underlying the observed changes in diffusion tensor characteristics (e.g., *is a change in FA driven by a change in axonal density, axonal orientation configuration or both?)*, striving to improve the biological specificity of diffusion MRI.

### Limitations

Firstly, we acknowledge that while both NODDI and SMT may be able to detect alterations due to microstructural pathology, they both provide estimates of neuronal tissue properties that are subject to biases, and therefore should be always interpreted with care (39, 40), especially in grey matter, where model biases and inaccuracies are the strongest (39, 41).

Secondly, our healthy control group was also not age-matched to the patient group, with a mean difference of around 10 years. In studies on the effects of increasing age on WM microstructure, widespread reductions in FA are observed to occur from early adulthood (42, 43). As both increases in orientation dispersion and reductions in intra-neurite volume can cause reductions in FA, this effect represents a potential confounding factor in this study. For this reason, we have included age as a regressor in all our MS-control comparisons and EDSS regressions (i.e., for all DTI, NODDI and SMT metrics), and made sure to report differences that appear to be statistically independent of age (Table 2 and Figure 5). Nonetheless, we acknowledge that further, non-linear effects may persist and influence our results (44, 45).

Finally, we acknowledge that several other different models could have been included in this comparison. We justify our choice of focussing on NODDI by pointing out that it is one of the most popular models for clinically feasible neurite density and morphology mapping, with a considerable literature establishing its use in MS (20, 21, 46). Similarly, SMT has been previously used in MS (25, 47) measures similar biophysical features and offers theoretical methodological advantages over NODDI. It is therefore scientifically relevant to investigate how its metrics relate to NODDI in the context of MS, at least in this first, exploratory comparison. Results from this study inform MS neurologists about the agreement of such popular techniques, providing them with useful information to interpret results that use either of the two techniques. In future, we plan to include additional techniques in similar comparisons (e.g., DKI, map-MRI).

## Conclusions

To conclude, both NODDI and SMT detect white matter microstructural differences between MS patients and controls, showing alterations in indices representing the underlying density and orientation dispersion of neurites that correlate with disability. Importantly, NODDI and SMT metrics with similar biophysical interpretation are strongly correlated among each other, and provide results that go in the same direction, giving confidence to the comparability of findings between studies using these two techniques.

## Data Availability Statement

The raw data supporting the conclusions of this article will be made available by the authors, without undue reservation.

## Ethics Statement

The study was reviewed and approved by NHS Health Research Authority, NRES Committee London - City Road & Hampstead, References: 135700-CIS2014 (13/LO/1413), 13/0231-CIS2013 (13/LO/1762). The patients/participants provided their written informed consent to participate in this study.

## Author Contributions

DJ: data analysis and experiment design. AR: data analysis/presentation and editor. WB and SC: image preparation, editor, and data acquisition. BK and FP: image preparation, editor, and data analysis. EK, AT, and DA: editor and experiment design. CG and OC: supervisor, senior editor, and experiment design. FG: data analysis/presentation, editor, and experiment design. All authors contributed to the article and approved the submitted version.

## Conflict of Interest

FG is employed by the Vall d'Hebron Institute of Oncology (Barcelona, Spain) in a study funded by AstraZeneca (PREdICT). AstraZeneca was not involved in the study design, collection, analysis, interpretation of data, the writing of this article or the decision to submit it for publication. The remaining authors declare that the research was conducted in the absence of any commercial or financial relationships that could be construed as a potential conflict of interest.
