# Supplementary material for: Comparison of Neurite Orientation Dispersion and Density Imaging and Two-Compartment Spherical Mean Technique Parameter Maps in Multiple Sclerosis
Source: Front Neurol. 2021 Jun 14;12:662855. doi: 10.3389/fneur.2021.662855 (PMC8236830; doi:10.3389/fneur.2021.662855)
Supplement: Supplementary file 1 [file Image_1.PDF]

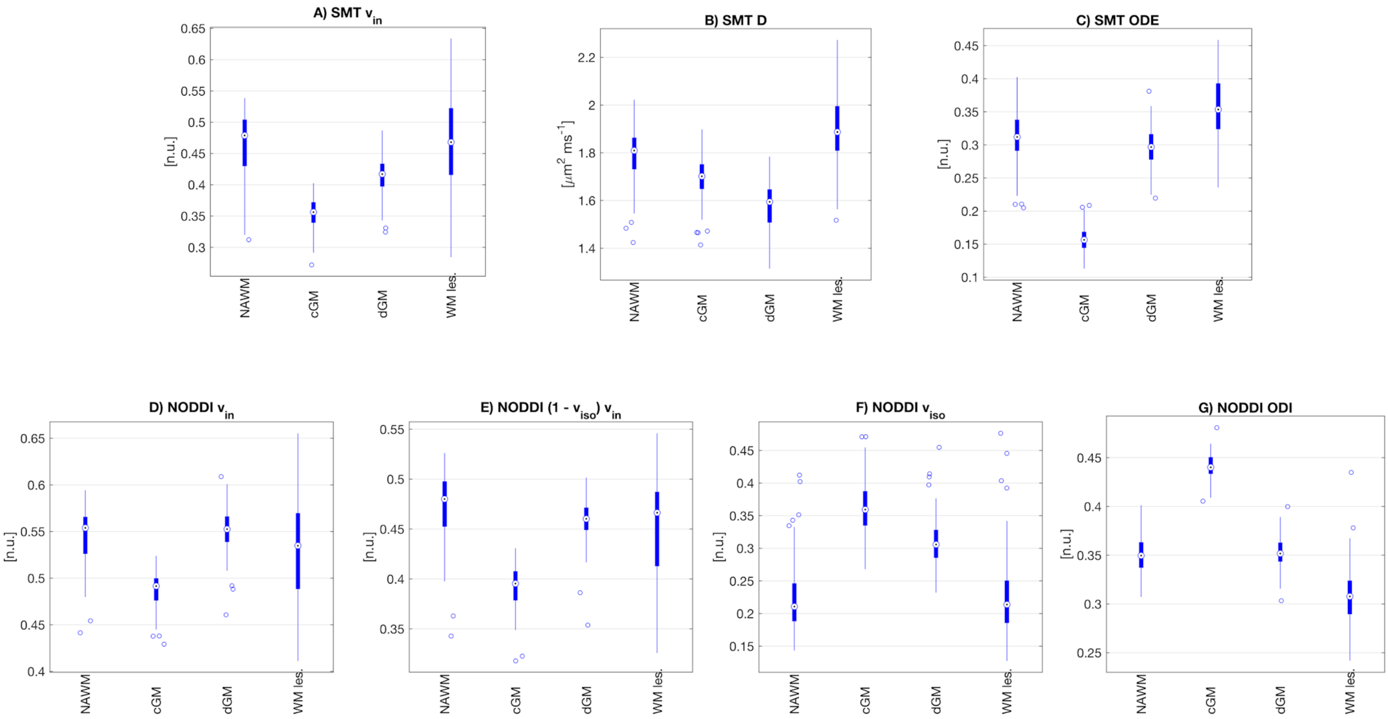

Supplementary Figure 1. Tissue-specific distributions of NODDI and SMT metrics in MS patients. Horizontal axis abbreviations: NAWM – as text; dGM - deep grey matter; cGM - cortical grey matter, les. WM - lesioned white matter. Top (A to C): SMT (intra-neurite volume fraction vin in A, neural diffusivity in B, orientation dispersion entropy ODE in C); bottom (D to G): NODDI (intra-neurite volume fraction vin in D; intra-neurite volume fraction corrected for isotropic volume fraction viso in E; isotropic volume fraction viso in F; orientation dispersion index ODI in G).
