# Supplementary material for: Comparison of Neurite Orientation Dispersion and Density Imaging and Two-Compartment Spherical Mean Technique Parameter Maps in Multiple Sclerosis
Source: Front Neurol. 2021 Jun 14;12:662855. doi: 10.3389/fneur.2021.662855 (PMC8236830; doi:10.3389/fneur.2021.662855)
Supplement: Supplementary file 2 [file Image_2.PDF]

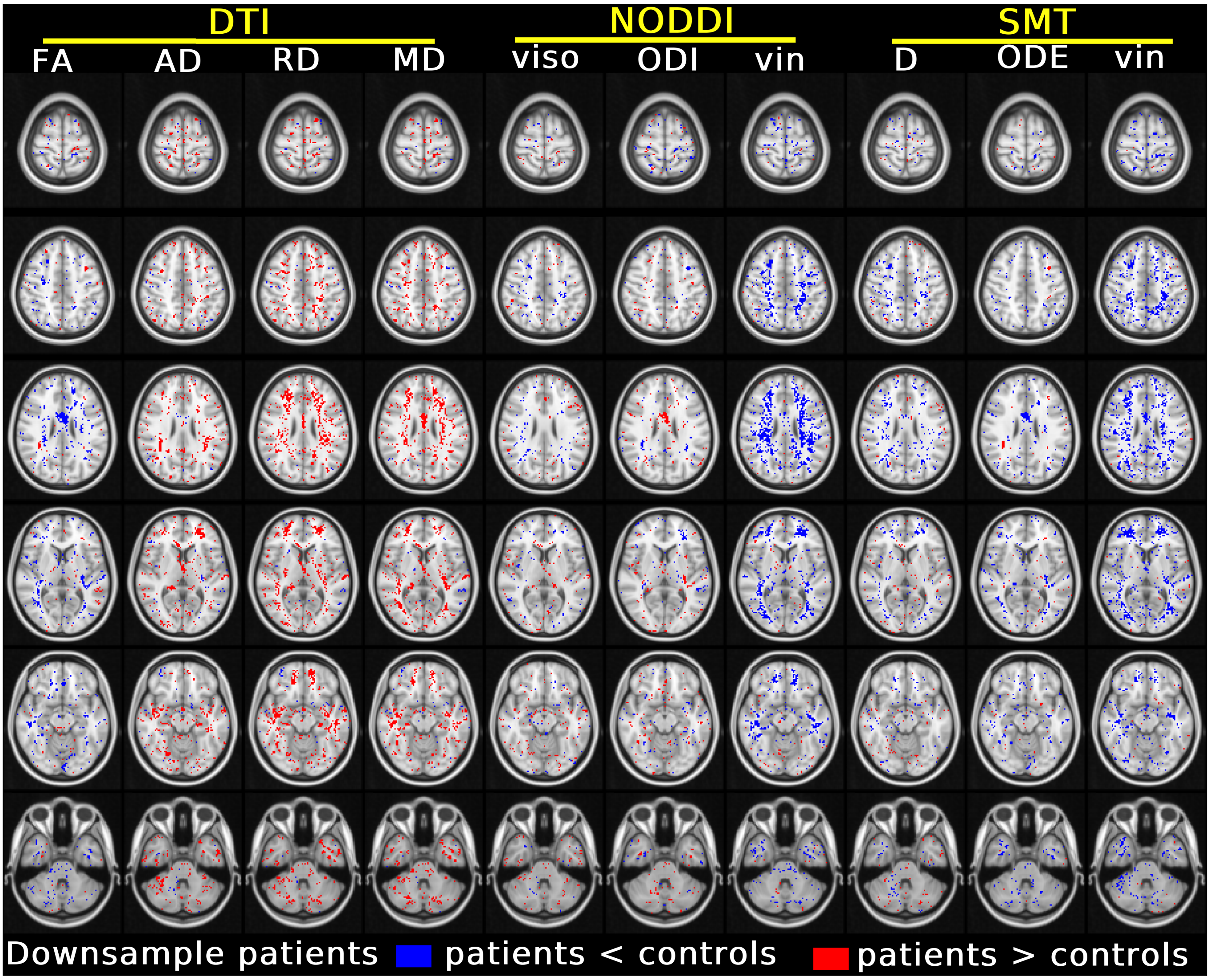

Supplementary Figure 2. Results of the voxel-wise group comparison performed in MNI space when matching the sizes of the patient/control groups by downsampling the patient group. The figure visualises voxels at 6 different axial levels where a specific MRI metric from DTI, NODDI and SMT differs significantly between patients and controls (blue/red: metric from patients smaller/larger than controls). The model used for this comparison, in the form of $m = \beta_0 + \beta_1 group + \beta_2 age + \beta_3 gender$ with m being the generic MRI metric, adjusts for age and gender. In each MNI voxel, the model was fitted excluding measurements from focal lesions (i.e., including only normal-appearing tissues). A threshold of $p < 0.05$, correcting for multiple comparisons with the FSL *fdr* tool, was chosen for statistical significance. The figure shows voxels where β_1 is statistically different from 0.
